# Propofol suppresses microglial phagocytosis through the downregulation of MFG-E8

**DOI:** 10.1186/s12974-020-02061-3

**Published:** 2021-01-09

**Authors:** Xiaoying Cai, Ying Li, Xiaoyang Zheng, Rong Hu, Yingyuan Li, Liangcan Xiao, Zhongxing Wang

**Affiliations:** Department of Anesthesiology, The First Affiliated Hospital, Sun Yat-sen University, No. 58 Zhongshan 2nd Road, Guangzhou, 510080 Guangdong People’s Republic of China

**Keywords:** Propofol, Microglia, MFG-E8, Phagocytosis

## Abstract

**Background:**

Microglia are highly motile phagocytic cells in the healthy brain with surveillance and clearance functions. Although microglia have been shown to engulf cellular debris following brain insult, less is known about their phagocytic function in the absence of injury. Propofol can inhibit microglial activity, including phagocytosis. Milk fat globule epidermal growth factor 8 (MFG-E8), as a regulator of microglia, plays an essential role in the phagocytic process. However, whether MFG-E8 affects the alteration of phagocytosis by propofol remains unknown.

**Methods:**

Microglial BV2 cells were treated with propofol, with or without MFG-E8. Phagocytosis of latex beads was evaluated by flow cytometry and immunofluorescence. MFG-E8, p-AMPK, AMPK, p-Src, and Src levels were assessed by western blot analysis. Compound C (AMPK inhibitor) and dasatinib (Src inhibitor) were applied to determine the roles of AMPK and Src in microglial phagocytosis under propofol treatment.

**Results:**

The phagocytic ability of microglia was significantly decreased after propofol treatment for 4 h (*P* < 0.05). MFG-E8 production was inhibited by propofol in a concentration- and time-dependent manner (*P* < 0.05). Preadministration of MFG-E8 dose-dependently (from 10 to 100 ng/ml) reversed the suppression of phagocytosis by propofol (*P* < 0.05). Furthermore, the decline in p-AMPK and p-Src levels induced by propofol intervention was reversed by MFG-E8 activation (*P* < 0.05). Administration of compound C (AMPK inhibitor) and dasatinib (Src inhibitor) to microglia blocked the trend of enhanced phagocytosis induced by MFG-E8 (*P* < 0.05).

**Conclusions:**

These findings reveal the intermediate role of MFG-E8 between propofol and microglial phagocytic activity. Moreover, MFG-E8 may reverse the suppression of phagocytosis induced by propofol through the regulation of the AMPK and Src signaling pathways.

## Introduction

Microglia, resident macrophages of the central nervous system (CNS), account for 5–12% of brain cells [1] and mediate principal immune activities [2]. They can become activated in response to some pathological stimuli, such as exogenous agents and apoptotic or necrotic cells. When not activated, microglia, which are emerging as important regulators during brain development, exist in a resting state under physiological conditions [3]. Resting microglia are responsible for continuous immune surveillance, sense subtle changes in the microenvironment, and perform subsequent actions. For example, when microglia encounter synaptic debris, cells perform phagocytosis by targeting debris, playing a crucial role in synaptic pruning in the postnatal brain [4]. Disruption of microglial phagocytosis impairs the above physiological process. Previous studies have found that microglial clearance ability might be influenced by some medical agents, leading to subsequent neurological disorders [5, 6]. This evidence highlights the importance of the phagocytic function of resting microglia in maintaining CNS homeostasis and suggests that the mechanisms of phagocytosis may represent possible therapeutic targets.

Propofol, a widely used short-acting intravenous sedative agent used in clinical practice, is often selected for general anesthesia or sedation of patients receiving surgery or undergoing diagnostic procedures [7]. Evidence has indicated that propofol can attenuate microglial responses when cells are exposed to external stimuli [8, 9]. It can inhibit pressure-stimulated macrophage phagocytosis via the GABA_A_ receptor [10]. However, there is limited evidence concerning the effect of propofol on microglia phagocytosis under resting conditions, in which inflammation and other stimulating factors do not surround microglial cells. Hsing et al*.* found that a high dose of propofol (140 μM) suppresses the phagocytic ability of BV2 cells [11]. Chen et al*.* revealed that propofol inhibits the phagocytosis of *Staphylococcus aureus*-stimulated RAW264.7 cells [12]. According to these studies, a high dose of propofol can attenuate microglial/macrophage phagocytosis. However, it represents a drug abuse scenario, whereas the effects of the more common levels of clinical propofol exposure remain unexplored. Additionally, the underlying mechanisms are as of yet unclear. Thus, it is important to explore the potential changes in microglial phagocytosis and identify the appropriate modulatory intermediate targets.

Milk fat globule epidermal growth factor 8 (MFG-E8), also called lactadherin, is secreted by dendritic cells, macrophages, and epithelial cells [13]. The N-terminal EGF-like domain of MFG-E8 can recognize the α_v_β_3_/α_v_β_5_-integrin macrophage receptor, and its C-terminal discoidin-like domain can recognize phosphatidylserine (PS) on the cell membranes of apoptotic cells/debris. It then acts as a bridge between apoptotic cells/debris and macrophages, connecting the integrin receptor and PS and facilitating the process of phagocytosis [14]. Recently, its role in modulating microglia has attracted increasing attention. Our previous study suggested that MFG-E8 participates in the alteration of M1/M2 polarization, and M1 and M2 microglia present different phagocytic tendencies [15]. Researchers have also suggested that microglia from MFG-E8 knockout animals exhibit deficits in phagocytic activity [16]. Considering that microglial phagocytosis ability is inhibited by propofol, we hypothesize that this process might be regulated through MFG-E8. Thus, we speculated that there is a possible link between MFG-E8 and propofol, and we conducted this study to investigate this link and its underlying mechanisms.

## Methods

### Materials

Propofol (D126608) and carboxylate-modified polystyrene yellow-green fluorescent latex beads (L4655) were purchased from Sigma-Aldridge Chemical Company (St. Louis, USA). Texas Red-X phalloidin (T7471) and DAPI (D1306) were purchased from Thermo Fisher Scientific (Waltham, USA). Mouse recombinant MFG-E8 protein (2805-MF-050/CF) and goat anti-mouse MFG-E8 antibody (MAB2805) were obtained from R&D Systems (Minneapolis, USA). MFG-E8-neutralizing antibody (sc-377356) and normal mouse IgG (sc-2025) were obtained from Santa Cruz Biotechnology (Santa Cruz, CA). Cytochalasin D (B6645) was purchased from Apexbio (Houston, USA). Compound C (S7306) and dasatinib (S1021) were purchased from Selleckchem (Houston, USA). F12-Dulbecco’s modified Eagle’s medium (DMEM, 10565018), fetal bovine serum (FBS, 10099141), phosphate-buffered saline (PBS, 10010023), and penicillin/streptomycin (15070063) were obtained from Life Technologies (New York, USA). Phospho-AMPKα (2535), AMPKα (5831), phospho-Src (6943), and Src antibodies (2108) were purchased from Cell Signaling Technology (Beverly, USA). MFG-E8 ELISA kit (EK1194) was purchased from Boster (Wuhan, China). GAPDH antibodies (ab8245) were purchased from Abcam (Cambridge, UK).

### Cell culture

BV2 cells were purchased from the China Academia Sinica Cell Repository (Shanghai, China). They were cultured using F-12 DMEM supplemented with 10% FBS and incubated at 37 °C in an atmosphere of 5% CO_2_ before treatment.

### CCK-8

Microglia were seeded in 96-well culture plates at a density of 2 × 10^4^ cells/well in 100 μl for 48 h to achieve a resting state. The medium was then replaced after culture. Cell proliferation was determined by the CCK-8 assay kit. In addition, 1‰ DMSO was used as a control. The absorbance of the solution was measured with a SpectraMax M5 multimode microplate reader at 450 nm. Cell viability is expressed as a percentage relative to that of the control group.

### Western blot

BV2 cells were scraped and lysed in buffer [150 mM NaCl, 1 mM EGTA, 1 mM EDTA, 1 mM Na_3_VO_4_, 2.5 mM sodium pyrophosphate, 1% Triton X-100, 1 mM β-glycerophosphate, 20 mM Tris-HCl (pH 7.4), and protease inhibitors (1:1000)] for ≥ 30 min. Total intracellular protein was extracted and quantified by a BCA kit according to the standard protocol of the manufacturer. The cell lysates were solubilized with SDS sample buffer (20 μg/lane) and separated by 10% SDS-PAGE (110 V, 75 min). The proteins were then transferred to a 0.45-μm PVDF membrane at 100 V for 1.5 h. After that, the membrane was blocked using TBST with 5% milk. Following incubation with antibodies (anti-MFG-E8, 1:500; anti-p-AMPKα, 1:1000; anti-AMPKα, 1:1000; anti-p-Src, 1:1000; anti-Src, 1:1000; and anti-GAPDH, 1:5000) for 12 h at 4 °C, the membrane was incubated with horseradish peroxidase (HRP)-conjugated secondary antibodies for 2 h at room temperature. After the membrane was washed with TBST, the immunoreactive bands were visualized by an enhanced chemiluminescence (ECL) plus detection system (Merck Millipore, USA) and scanned by the ImageQuant LAS 4000 system. The density of each band was quantified with the ImageJ software. The expression ratio was defined as the intensity of each band relative to that of GAPDH and normalized to the relative band intensity of the control group.

### Elisa

The microglial culture medium was replaced, serum-free culture media was added, and the microglia were cultured further. After treatment, supernatants were collected from each group and evaluated in duplicate using ELISA kits in accordance with the standard protocol. After the standard samples were prepared, the experimental samples were incubated for 30 min at 37 °C. After repeated washings in PBS, ELISA reagents were added, and the samples were incubated for 30 min incubation at 37 °C. Finally, developing solution was added, and the absorbance of each well was measured at 450 nm with a microplate reader. The average absorbance values of each set of standards and samples were calculated, and a standard curve was constructed. The concentrations of the samples were calculated from the standard curve.

### Immunofluorescence

Microglia were seeded at a density of 2 × 10^4^ cells/well on 1.5-mm^2^ coverslips for 24 h before treatment. Then, the cell culture medium was removed. The cells were fixed with 4 °C 4% paraformaldehyde for 30 min. After that, they were gently washed with PBS, blocked, and permeabilized with 5% BSA in PBS containing 0.3% Triton for 1 h. The samples were incubated with Texas Red-X phalloidin (1:500) for 30 min. The coverslips were transferred onto glass slides after 5 min of staining with DAPI. Images were observed and captured under an Olympus BX-51 microscope (Olympus, Tokyo, Japan).

### Phagocytosis assay

Microglial phagocytosis was evaluated by flow cytometry and immunofluorescence.

For flow cytometry, microglia were plated in 35-mm dishes at a density of 2 × 10^5^ cells/dish for 24 h. After treatment, fluorescence-labeled latex beads were added at a concentration of 1.12 μl/ml for 45 min at 37 °C. Then, cells were washed three times with PBS to remove the non-phagocytized beads, trypsinized and harvested. Cells were resuspended in 4% paraformaldehyde, and phagocytosis of the beads by microglia was detected by flow cytometry (Beckman Coulter CytoFLEX). The data were analyzed by using the CytExpert software to evaluate phagocytosis of the beads by microglia.

For immunofluorescence, microglia were plated at a density of 2 × 10^4^ cells/well on 1.5-mm^2^ coverslips for 24 h. Then, fluorescence-labeled latex beads were added at a concentration of 5 μl/ml for 2 h at 37 °C. The cells were washed three times with PBS to remove the non-phagocytized beads and fixed with 4% paraformaldehyde. Next, phagocytosis of the beads by microglia was observed under a fluorescence inverted microscope. Cells with phagocytic activity were observed using a fluorescence microscope (× 40 and × 100 magnification). In addition, the amount of latex beads phagocytized by microglia was detected by flow cytometry. All experiments were repeated three times. Microglia in the negative control group were pretreated with cytochalasin D (10 μM) for 30 min.

### Statistical analysis

Data were presented as the mean ± standard derivation (SD) and were analyzed with the Statistical Package for Social Science (SPSS) 24.0 software. One-way analysis of variance (ANOVA) followed by the least significant difference (LSD) test was used to analyze the variables. The level of statistical significance was set at *P* < 0.05.

## Results

### Microglia phagocytosis in response to propofol

**Fig. 1:**
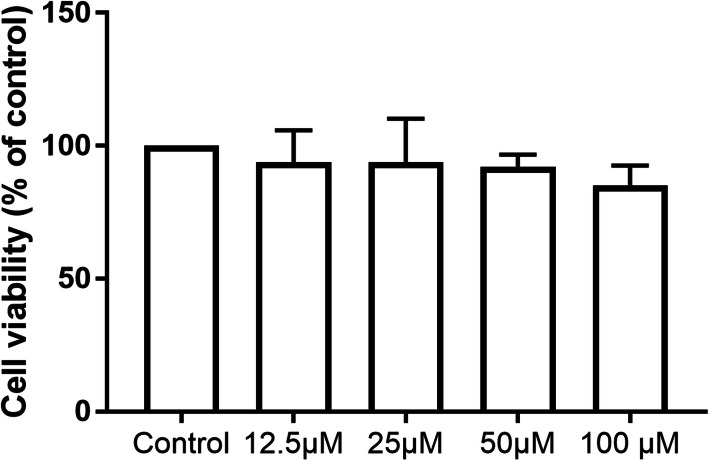
Microglial viability after propofol pretreatment. The viability of BV2 cells after treatment with propofol (12.5 μM, 25 μM, 50 μM, or 100 μM) for 4 h was measured by the CCK-8 assay

**Fig. 2:**
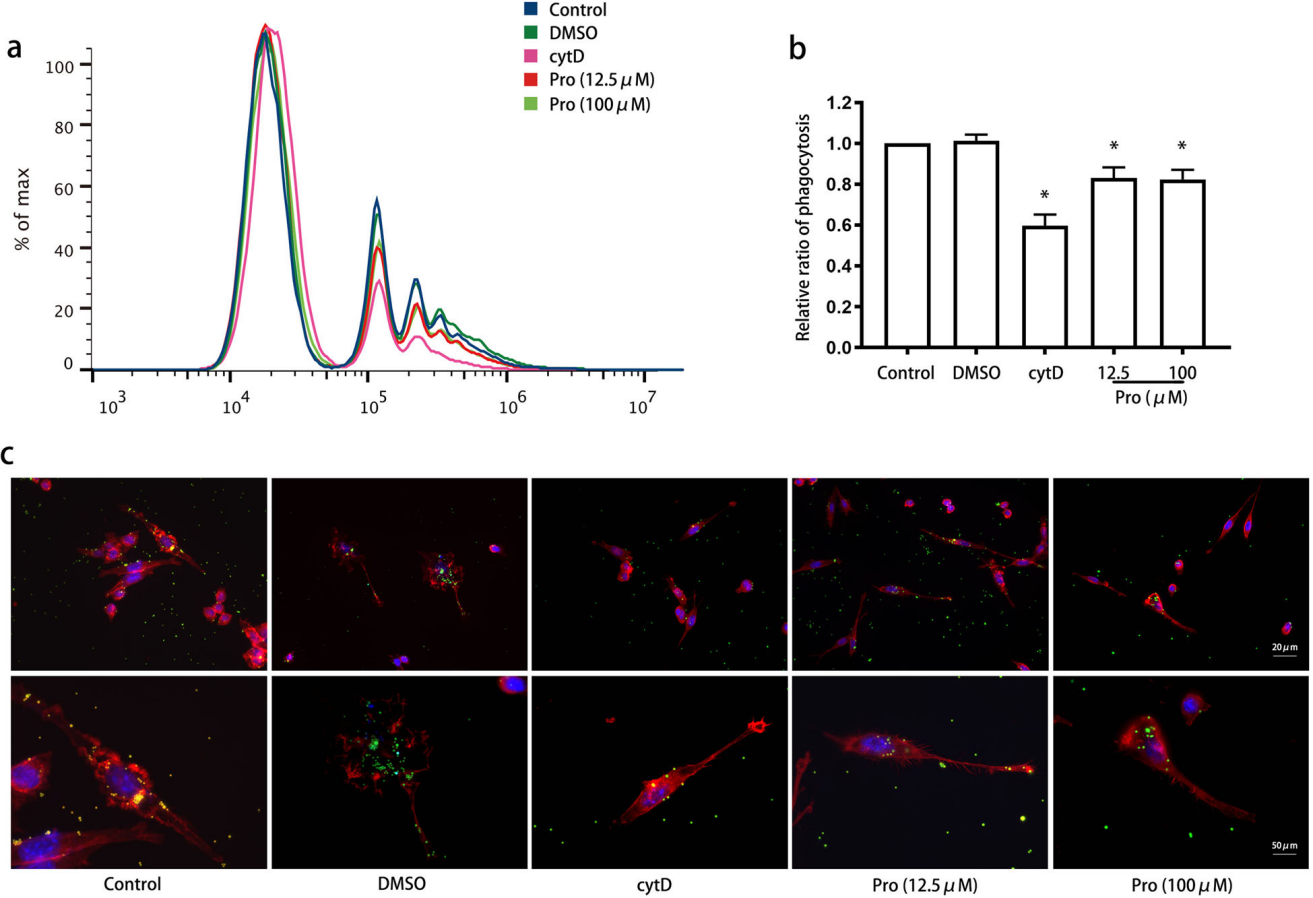
Propofol attenuated BV2 cell phagocytosis in a dose-dependent manner. BV2 cells were treated with propofol (12.5 μM or 100 μM), and phagocytic ability was measured using flow cytometry (**a**, **b**) and immunofluorescence (**c**). Immunofluorescence staining: phalloidin-red, latex beads-green, DAPI-blue. The data are presented as the mean ± SD. Pro, propofol; cytD, cytochalasin D. *P < 0.05 versus DMSO

The viability of BV2 cells was measured after treatment with propofol (12.5 μM, 25 μM, 50 μM, and 100 μM) for 4 h (Fig. 1). Our results showed that propofol did not significantly influence cell viability, compared with control (*P* > 0.05). Furthermore, we evaluated the effects of propofol on the innate phagocytic activity of microglia. Flow cytometry indicated that fewer latex beads were engulfed by cells treated with propofol than those treated with the control, and this effect of propofol on phagocytosis was dose-dependent from 12.5 to 100 μM (*P* < 0.05) (Fig. 2a, b). The microanatomical details of the microglia-latex bead interactions were observed by immunofluorescence (Fig. 2c). Propofol-treated cells exhibited a smaller cell body with fewer terminal tips and fewer beads inside than control-treated cells. The size and number of terminals were negatively correlated with the propofol concentration.

**Fig. 3:**
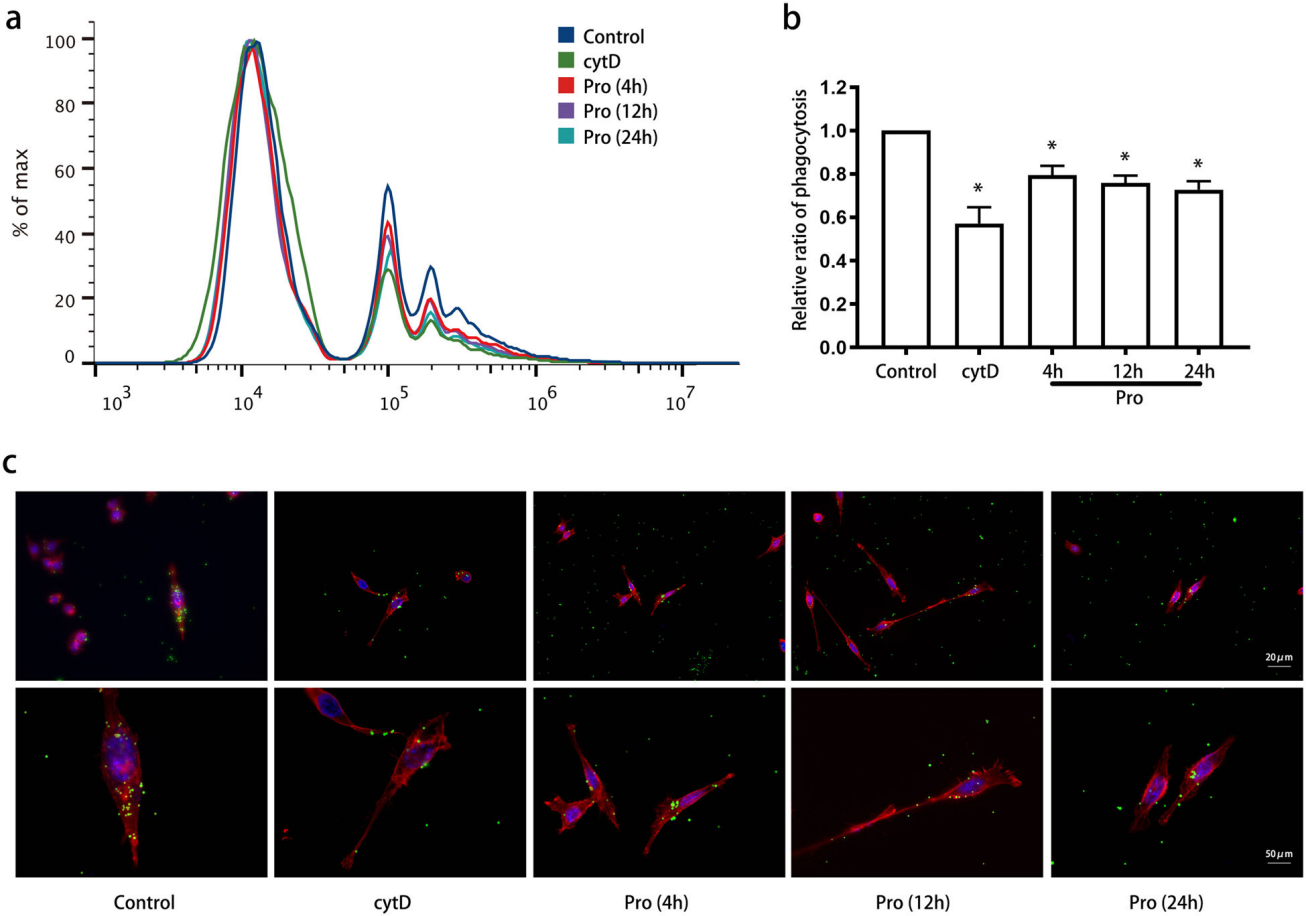
Propofol attenuated BV2 cell phagocytosis in a time-dependent manner. BV2 cells were treated with propofol (12.5 μM), and phagocytic ability was measured at 4 h, 12 h, and 24 h using flow cytometry (**a**, **b**) and immunofluorescence (**c**). Immunofluorescence staining: phalloidin-red, latex beads-green, DAPI-blue. The data are presented as the mean ± SD. Pro, propofol; cytD, cytochalasin D. * *P* < 0.05 versus the control

Then, we examine the time-dependent effect of 12.5 μM propofol based on the abovementioned findings. This dose of propofol is closer to the effect compartment concentration (2.2 μg/mL) widely used in clinical practice. Microglial phagocytosis was significantly reduced by 12.5 μM propofol, with phagocytic activity plateauing at 4 h (Fig. 3a, b). Additionally, propofol-treated cells were smaller and exhibited fewer branches than control-treated cells (Fig. 3c).

### MFG-E8 production by microglia in response to propofol

**Fig. 4:**
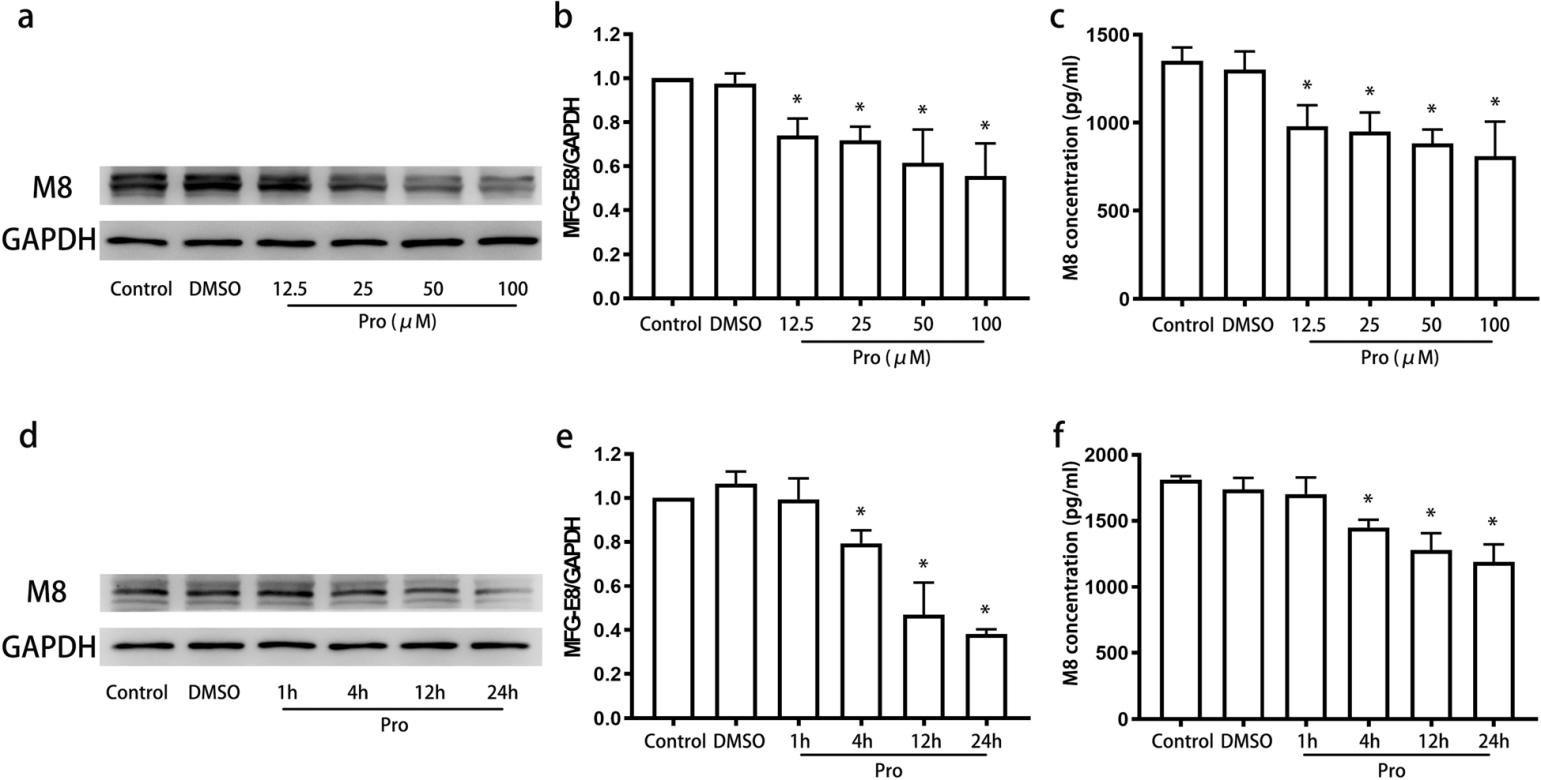
Microglial MFG-E8 expression in response to propofol. BV2 cells were treated with propofol (12.5 μM, 25 μM, 50 μM, or 100 μM) for 4 h (**a**, **b**, **c**). Then, microglia were treated with propofol (12.5 μM) for 1 h, 4 h, 12 h, or 24 h (**d**, **e**, **f**). Representative western blot images are displayed in **a** and **d**. Quantitative analysis of the western blot data is shown in **b** and **e**. The expression of MFG-E8 in the supernatant was measured by ELISA (**c**, **f**). The data are presented as the mean ± SD. Pro, propofol. *P < 0.05 versus DMSO

To evaluate the alteration in MFG-E8 levels, we measured the production of MFG-E8 by microglia 4 h after incubation with propofol (12.5 μM, 25 μM, 50 μM, or 100 μM). The results showed that propofol inhibited MFG-E8 expression in the cell lysate (Fig. 4a, b) and supernatant (Fig. 4c) in a concentration-dependent manner (*P* < 0.05). We further evaluated the time course of MFG-E8 expression after propofol treatment (12.5 μM). At this concentration, propofol time-dependently suppressed MFG-E8 expression (Fig. 4d, e), with the difference in expression being significant from 4 h (*P* < 0.05). Similar results were obtained by ELISA (Fig. 4f) (*P* < 0.05).

### Effects of MFG-E8 on microglial phagocytosis

**Fig. 5:**
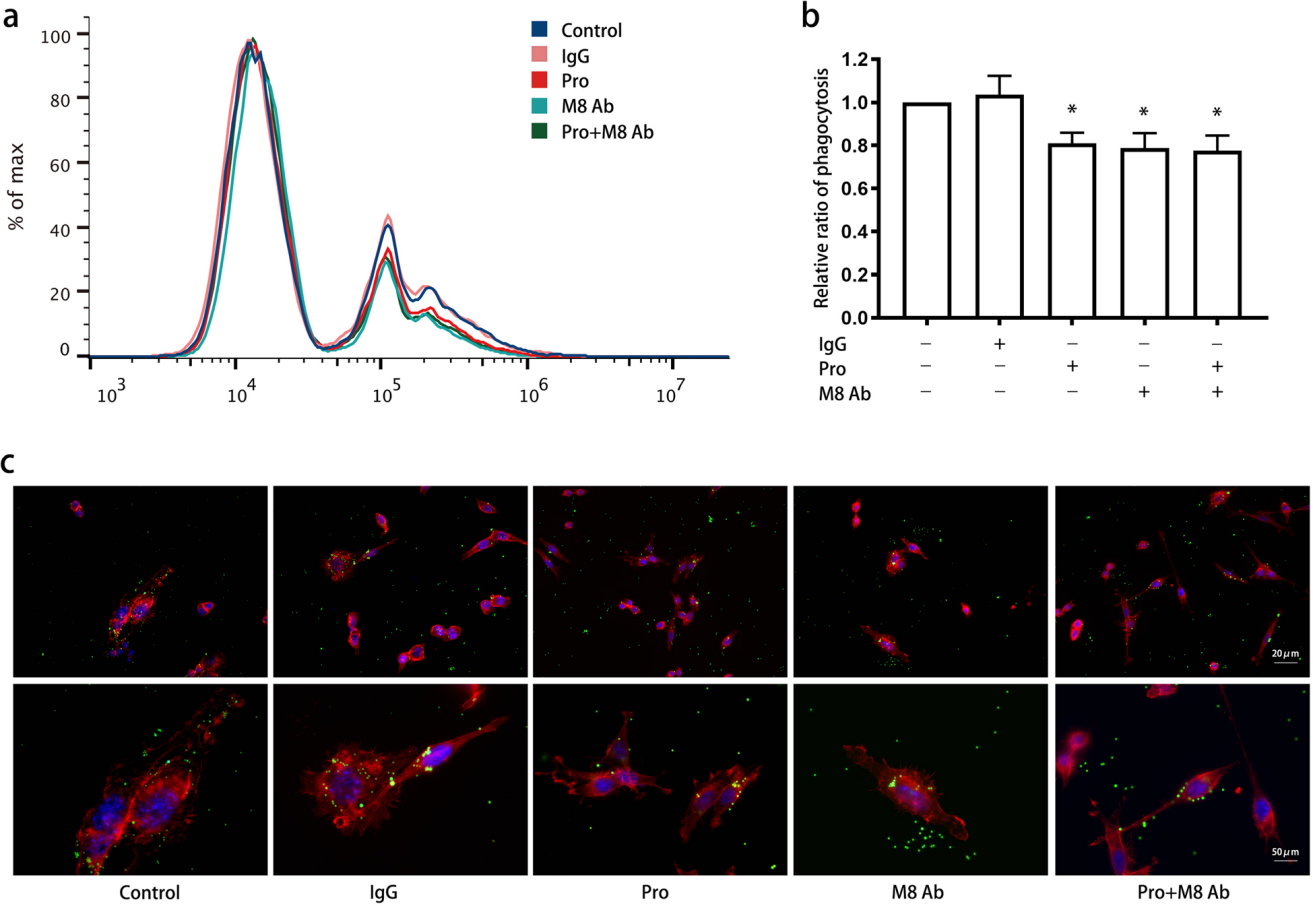
Blockage of MFG-E8 on the phagocytosis ability of microglia by propofol. BV2 cells were treated with or without an MFG-E8 antibody (5 μg/ml)/IgG antibody (5 μg/ml) for 45 min after treatment with propofol (12.5 μM) for 4 h. Flow cytometry (**a**, **b**) and immunofluorescence (**c**) were used to assess the microglial phagocytosis of latex beads. Immunofluorescence staining: phalloidin-red, latex beads-green, DAPI-blue. The data are presented as the mean ± SD. Pro, propofol; M8 Ab, MFG-E8-neutralizing antibody. *P < 0.05 versus the control

**Fig. 6:**
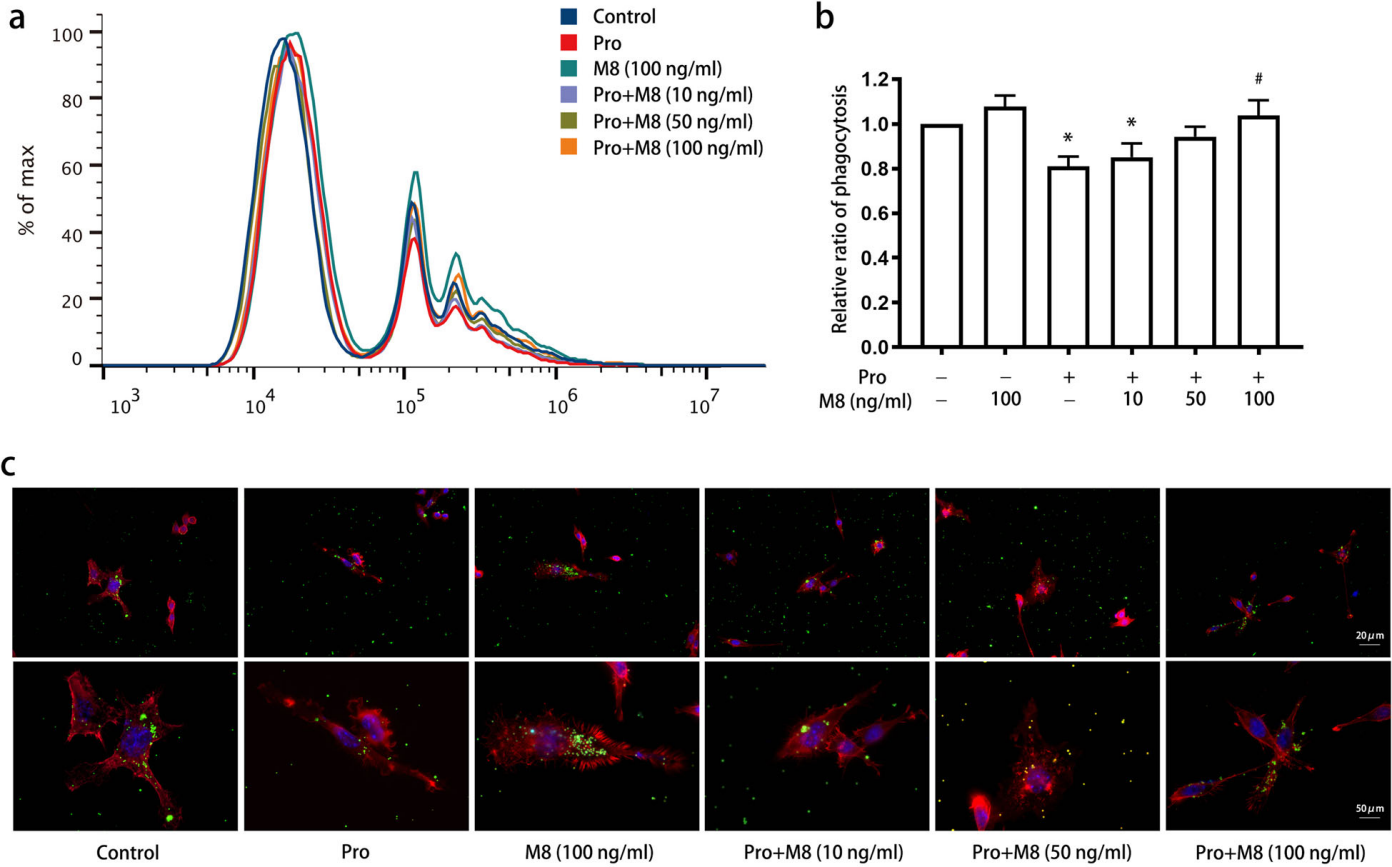
Incubation with MFG-E8 on the effect of propofol on microglial phagocytic ability. BV2 cells were treated with or without MFG-E8 (10, 50, or 100 ng/ml) for 45 min after treatment with propofol (12.5 μM) for 4 h. Flow cytometry (**a**, **b**) and immunofluorescence (**c**) were used to assess the microglial phagocytosis of latex beads. Immunofluorescence staining: phalloidin-red, latex beads-green, DAPI-blue. The data are presented as the mean ± SD. Pro, propofol; M8, MFG-E8. *P < 0.05 versus the control; ^#^*P* < 0.05 versus propofol

To validate the involvement of MFG-E8 in microglia phagocytosis, we first applied an MFG-E8-neutralizing antibody after propofol treatment. The antibody binds with MFG-E8 and blocks its functional activity. We found that a single application of the MFG-E8 antibody significantly inhibited the phagocytosis of latex beads (*P* < 0.05) (Fig. 5a, b) and decreased the number of terminals on and the body size of microglia (Fig. 5c). Propofol treatment followed by MFG-E8 antibody administration did not lead to changes in phagocytosis (*P* > 0.05). Furthermore, postadministration of MFG-E8 recombinant protein gradually reversed the suppression of phagocytosis by propofol as the concentration of the MFG-E8 recombinant protein increased from 10 to 100 ng/ml (Fig. 6a, b) (*P* < 0.05). Immunofluorescence indicated that microglia were larger and exhibited more terminal branches following treatment with the MFG-E8 recombinant protein (Fig. 6c). Although single administration of MFG-E8 (100 ng/ml) increased the phagocytosis rate, the difference was not significant (*P* > 0.05).

### Mechanism underlying the effect of MFG-E8 on propofol-induced alterations in microglial phagocytosis

**Fig. 7:**
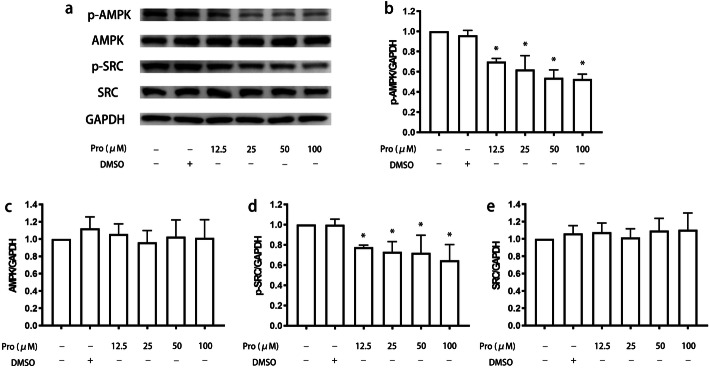
The expression of AMPK and Src in BV2 cells treated with propofol. BV2 cells were treated with propofol (12.5 μM, 25 μM, 50 μM, or 100 μM) for 4 h. The expression of p-AMPK, AMPK, p-Src, Src, and GAPDH was measured by western blotting (**a**). The bands were quantitatively analyzed (**b**, **c**, **d**, **e**). The data are presented as the mean ± SD. Pro, propofol. *P < 0.05 versus DMSO

**Fig. 8:**
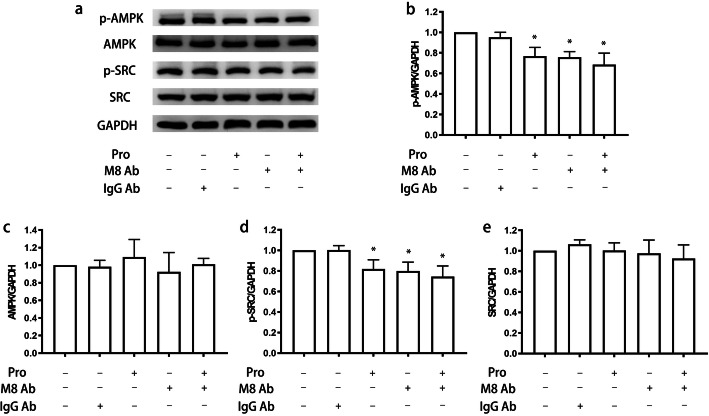
Blockage of MFG-E8 on the expression of AMPK and Src in microglia treated with propofol. BV2 cells were treated with or without an MFG-E8 antibody (5 μg/ml)/IgG antibody (5 μg/ml) for 45 min after treatment with propofol (12.5 μM) for 4 h. The expression of p-AMPK, AMPK, p-Src, Src, and GAPDH was measured by western blotting (**a**). The bands were quantitatively analyzed (**b**, **c**, **d**, **e**). The data are presented as the mean ± SD. Pro, propofol; M8 Ab, MFG-E8-neutralizing antibody. *P < 0.05 versus the control

**Fig. 9:**
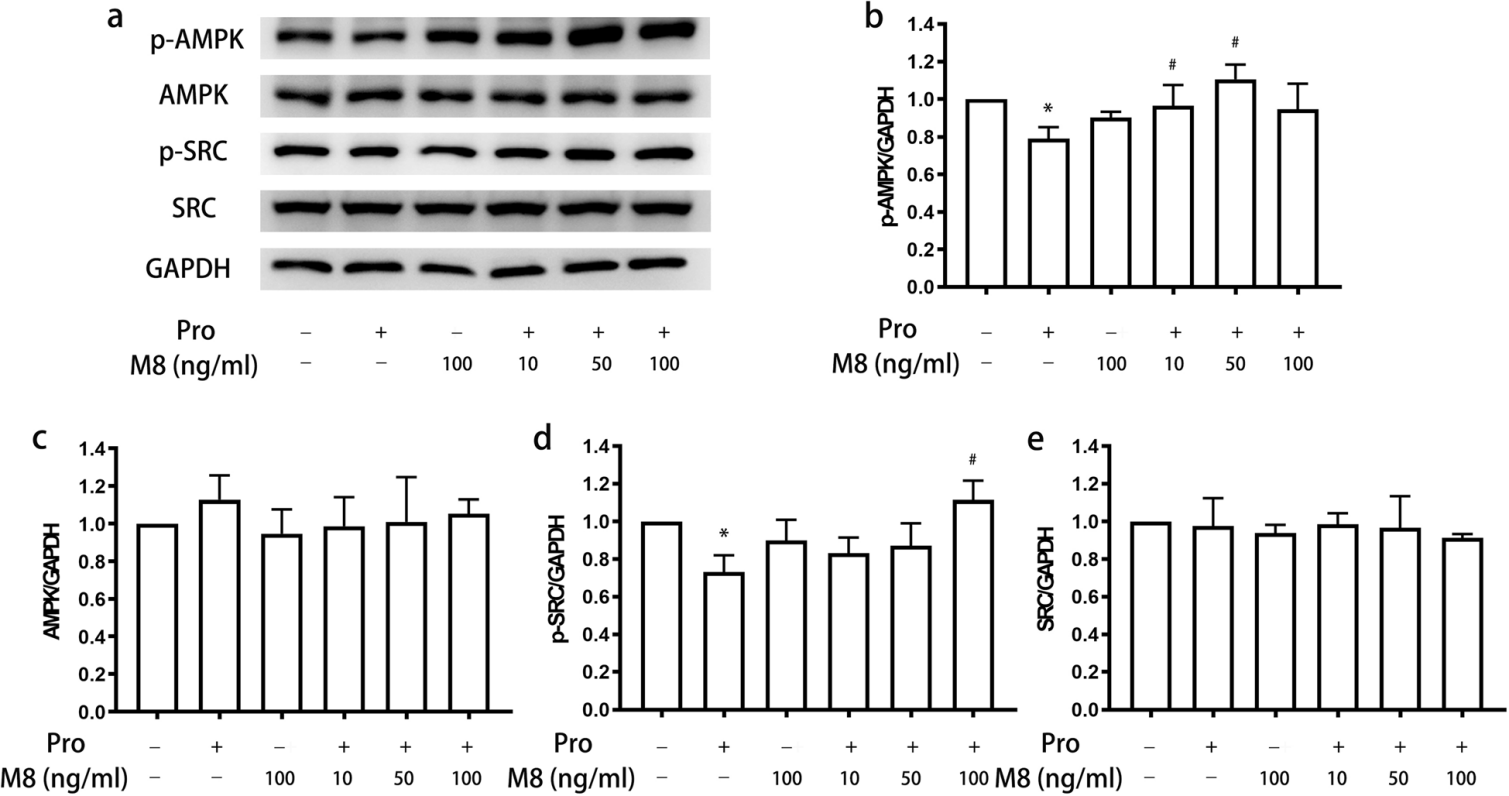
Effect of postadministration of MFG-E8 on the expression of AMPK and Src in microglia treated with propofol. BV2 cells were treated with or without MFG-E8 (10, 50, or 100 ng/ml) for 45 min after treatment with propofol (12.5 μM) for 4 h. The expression of p-AMPK, AMPK, p-Src, Src, and GAPDH was measured by western blotting (**a**). The bands were quantitatively analyzed (**b**, **c**, **d**, **e**). The data are presented as the mean ± SD. Pro, propofol; M8, MFG-E8. *P < 0.05 versus the control; ^#^*P* < 0.05 versus propofol

First, we treated microglia with propofol and found that it inhibited the levels of phospho-AMP-activated protein kinase (p-AMPK) and phospho-steroid receptor coactivator (p-Src) in a dose-dependent manner (*P* < 0.05) (Fig. 7). Specifically, p-AMPK expression reached a plateau after treatment with 50 μM propofol and remained stable following treatment with 100 μM propofol. Then, we attempted to validate the effects of MFG-E8 on the signaling pathway involving p-AMPK and p-Src. The results indicated that the MFG-E8 antibody had a similar suppressive effect on the expression of p-AMPK and p-Src as propofol (*P* < 0.05) (Fig. 8). We administered MFG-E8 after propofol treatment and found that MFG-E8 reversed propofol-induced inhibition of p-Src expression in a concentration-dependent manner (*P* < 0.05) (Fig. 9a, d). Similarly, p-AMPK expression reached a plateau following treatment with 50 ng/ml MFG-E8 antibody and remained stable following treatment with 100 ng/ml MFG-E8 antibody (*P* < 0.05) (Fig. 9a, b). However, single administration of the MFG-E8 antibody (100 ng/ml) did not alter the expression of either p-AMPK or p-Src (*P* > 0.05) (Fig. 9).

### Role of AMPK and Src in microglial phagocytosis

**Fig. 10:**
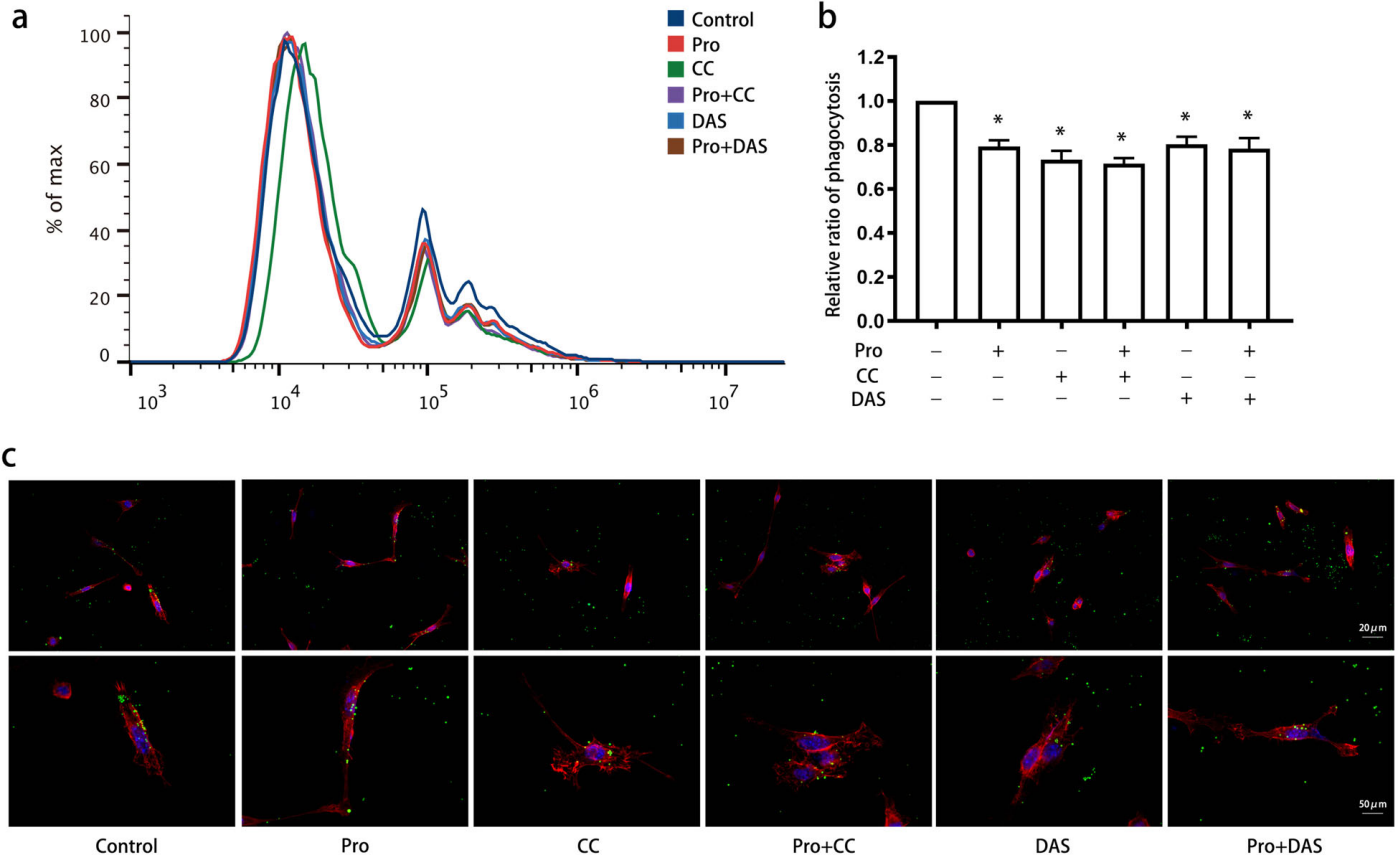
Involvement of the AMPK and Src pathways in the effects of propofol on microglial phagocytosis. BV2 cells were treated with or without dasatinib (100 nM, 30 min) and compound C (10 μM, 1 h) and treated with propofol. Flow cytometry (**a**, **b**) and immunofluorescence (**c**) were used to assess the microglial phagocytosis of latex beads. Immunofluorescence staining: phalloidin-red, latex beads-green, DAPI-blue. The data are presented as the mean ± SD. Pro, propofol; CC, compound C; DAS, dasatinib. *P < 0.05 versus the control

We administered compound C (AMPK inhibitor) and dasatinib (Src inhibitor) to microglia after propofol (12.5 μM) treated and monitored the phagocytosis of latex beads (Fig. 10). Marked inhibition of the engulfment of latex beads was observed in cells treated with compound C, dasatinib, and propofol (*P* < 0.05) (Fig. 10a, b). Immunofluorescence showed that cells treated with these agents exhibited smaller cell sizes and fewer branches than those treated with the control (Fig. 10c).

**Fig. 11:**
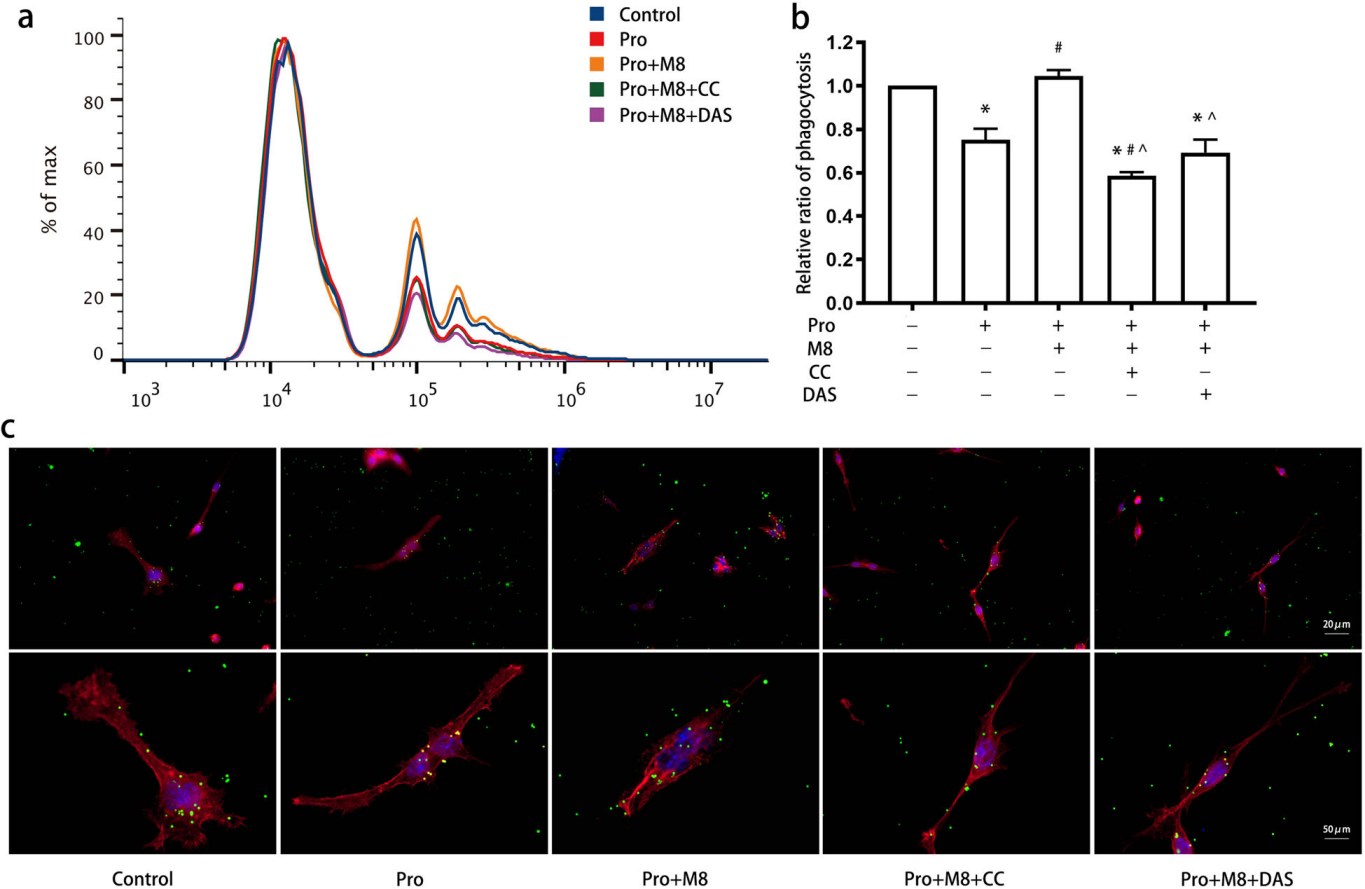
Effects of AMPK and Src pathway modulation on MFG-E8-mediated microglial phagocytosis. BV2 cells were treated with dasatinib (100 nM, 30 min) and compound C (10 μM, 1 h), treated with propofol, and then incubated with MFG-E8 (100 ng/ml) and fluorescence-labeled latex beads. Flow cytometry (**a**, **b**) and immunofluorescence (**c**) were used to assess the microglial phagocytosis of latex beads. Immunofluorescence staining: phalloidin-red, latex beads-green, DAPI-blue. The data are presented as the mean ± SD. Pro, propofol; M8, MFG-E8; CC, compound C; DAS, dasatinib. *P < 0.05 versus the control; ^#^*P* < 0.05 versus propofol; ^^^*P* < 0.05 versus propofol+MFG-E8

To illustrate whether AMPK and Src participate in MFG-E8-mediated processes, we further treated microglia with compound C and dasatinib before the application of MFG-E8. We found that compound C and dasatinib significantly inhibited the elevation of microglial phagocytosis induced by MFG-E8 incubation (*P* < 0.05) (Fig. 11a, b). The cells treated with compound C and dasatinib showed fewer branches and shrunken bodies than those treated with MFG-E8 (Fig. 11c).

## Discussion

**Fig. 12:**
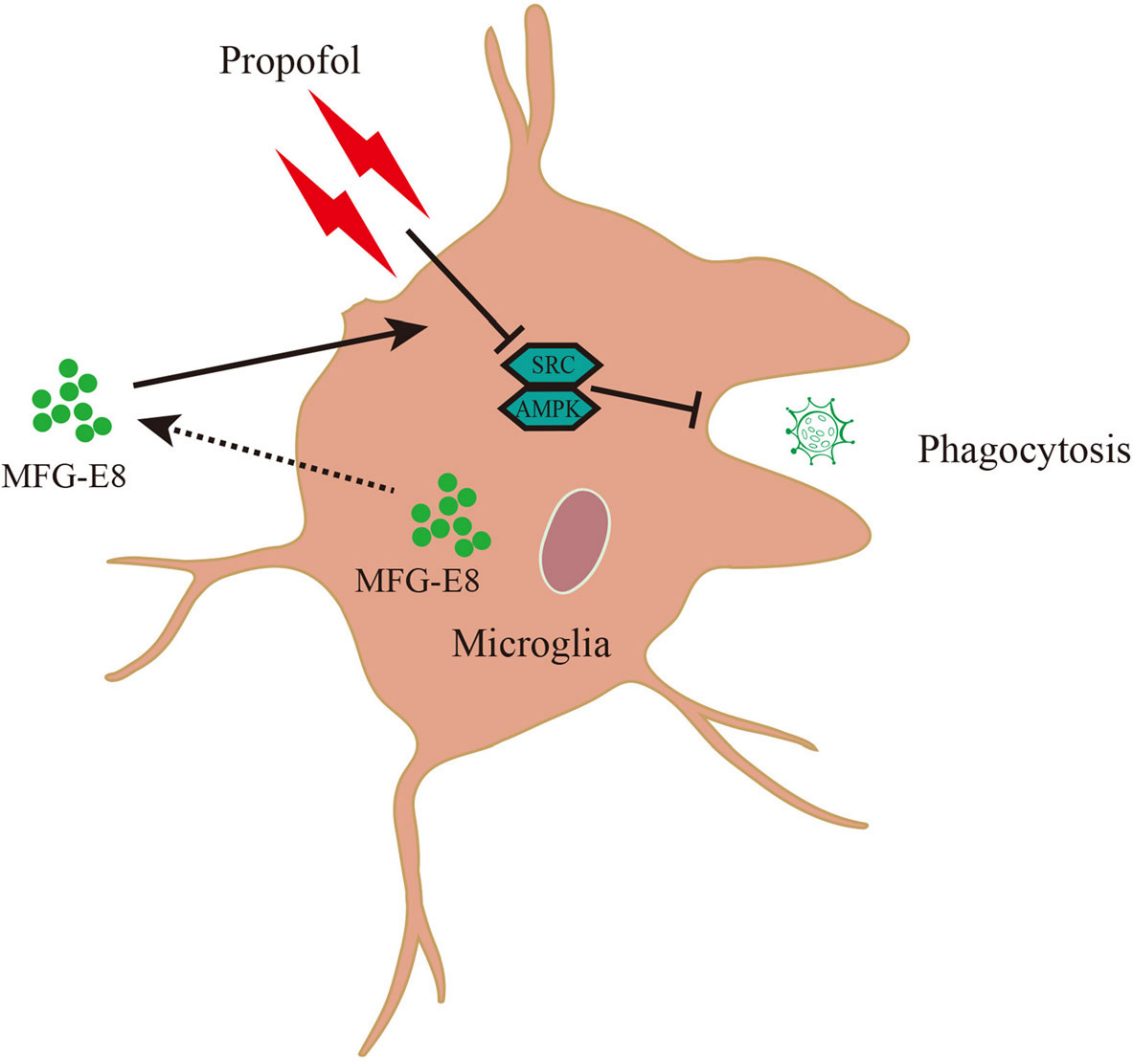
Schematic diagram for the effects of MFG-E8 on propofol-suppressed microglial phagocytosis. MFG-E8 restores the inhibited microglial phagocytic process by propofol, through the AMPK and Src signaling pathways

The current study reports that propofol reduces MFG-E8 production by microglia and may inhibit cellular phagocytosis in an MFG-E8-dependent manner. Moreover, the AMPK and Src pathways are required for the effects of MFG-E8 (Fig. 12, schematic diagram for the effects of MFG-E8 on propofol-suppressed microglial phagocytosis). Together, our findings identify the potential mechanism by which propofol affects microglial responses, especially phagocytic ability.

Current evidence indicates that microglial phagocytosis plays an important role in the homeostasis of the CNS. The benefits of the removal of debris and dead cells from the brain by microglia are extensive. A convincing example of these benefits is the prevention of Alzheimer’s disease development through the elimination of amyloid-β by microglia with the assistance of complement component C3 and complement receptor type 3 [17]. Previous studies have established the role of propofol in alleviating microglial activation. Chen et al. [12] showed that propofol inhibits reactive oxygen species (ROS) release and the phagocytic ability of macrophage RAW264.7 cells in response to infection. Yu et al. [6] revealed that propofol inhibited the microglial phagocytosis under increase pressure circumstance. In addition, Liu and his colleagues found that propofol can suppress microglial activity, including phagocytosis ability, in response to lipopolysaccharide (LPS) exposure [9]. However, there is currently limited evidence regarding the effect of a single administration of propofol on resting microglia, i.e., in the absence of activation-inducing insult. Propofol is sometimes used during surgery or imaging investigations for patients without systematic disorders, such as infection. Microglia in the brains of these patients are quite likely to indicate a non-neuroinflammatory condition. It is of great importance to understand the innate reactions that occur in the brain upon exposure to propofol only. Thus, in our study, we incubated microglia with propofol in the absence of any stimulating or inflammatory factors to mimic such conditions. We found that propofol inhibited the intrinsic abilities of microglia, which is consistent with previous findings, thus expanding our understanding of the effects of propofol [6, 10].

The toxic effects of propofol have been realized in recent years. Researchers have found that the neuronal viability is affected by propofol and that p38-mitogen-activated protein kinase (p38-MAPK) and extracellular signal-regulated kinase-1/2 (ERK1/2) are involved in neuronal death [18, 19]. However, we found that the viability of microglia is not affected by propofol. This finding implies that this agent can be used safely to modulate microglia-related functions, which is consistent with the findings of a previous study [20]. A possible explanation for the variation in the effect of propofol on cell viability could be differences in cell origin, as cells derived from different sources could show variations in sensitivity to stimuli. For example, neurons are vulnerable to external insults, including LPS exposure and oxygen-glucose deprivation (OGD) [21–23], while microglia show a much higher tolerance to environmental changes. Another possible explanation for the variation in the effect of propofol on the viability of neurons and microglia could be the heterogeneity of GABA_A_ receptor subunits within the brain [24]. Most of the actions of propofol result from its interaction with GABA_A_ receptors, which are expressed on neurons and glial cells. Previous studies have established that the canonical GABA_A_ receptors contain two α subunits, two β subunits, and a fifth subunit [25]. The diversity in subunit composition results in differences in agonist affinity for the receptor, the chance of opening, conductance, and other properties. These differences could be studied with newly established technology, such as clustered regularly interspaced short palindromic repeats/CRISPR-associated protein 9 (CRISPR/Cas9) approaches.

Various receptors and molecules participate in microglial phagocytosis. Among them, MFG-E8 has attracted increasing attention for its regulatory role in microglial activities. Our previous studies established the anti-inflammatory effect of MFG-E8 against microglia [15]. This molecule plays an essential role in orchestrating microglial activity, especially phagocytosis, through its interaction with α_V_β_3/5_ integrin. Brown et al. suggested that MFG-E8 is crucial for the maintenance of microglial phagocytosis in the presence of Aβ [14]. Neniskyte et al. demonstrated that microglia from MFG-E8 knockout mice do not exhibit phagocytic ability [16]. Thus, we speculated that the action of MFG-E8 is an important mechanism underlying the effects of propofol because MFG-E8 expression was decreased and the engulfment of latex beads was suppressed after propofol treatment in our study. Moreover, application of MFG-E8 reversed the impairment of phagocytosis induced by propofol, and this effect was blocked with a neutralizing antibody. These results indicate that propofol may inhibit microglial phagocytosis via the downregulation of MFG-E8 expression. Observation of the microglial cytoskeleton is important for studying phagocytic ability. We evaluated the cytoskeleton of microglia using immunofluorescence with Texas Red phalloidin. Cytoskeleton rearrangement reflects the amoeboid/phagocytic and branched/nonphagocytic statuses of microglia [26]. When microglia are transformed into a state with a high capacity for phagocytosis, they manifest an “amoeboid” morphology with a larger cell body and shorter, thick processes [27]. We provide the evidence that propofol-treated microglia are smaller and have fewer terminals than control-treated microglia, indicating that propofol has an inhibitory effect on the motility and rearrangement of the microglial cytoskeleton. MFG-E8 reversed these inhibitory effects, regulating the effect of propofol on the cytoskeleton.

The mechanism underlying cytoskeletal rearrangement after propofol stimulation remains unclear. In the present study, we studied the activity of the AMPK and Src pathways, which have been studied for their involvement in cellular cytoskeleton homeostasis [28]. AMPK is believed to be responsible for the phosphorylation of and conformational changes in cingulin, altering its binding with microtubules and actin filaments to regulate the cellular cytoskeleton [29]. AMPK activation enhances the phagocytic activity of macrophages, which might be suppressed by propofol [30, 31]. Src kinase has been revealed to be involved in the β2 integrin-related phagocytotic pathway. Deficiency of Src kinase could result in defective integrin-related phagocytic responses [32]. Moreover, a previous study indicated that upregulation of AMPK expression might occur through activation of Src [33]. Thus, we speculate that MFG-E8 mediates propofol-induced cytoskeleton regulation through the AMPK and Src pathways.

Decreased expression of p-AMPK and p-Src was observed after propofol treatment in this study, and a similar trend in MFG-E8 expression was observed, indicating a possible connection between MFG-E8 and these two signaling molecules. Subsequent treatment with MFG-E8 reversed the abovementioned alterations in p-AMPK and p-Src expression. This finding emphasizes the regulatory effect of MFG-E8 on AMPK and Src. The application of inhibitors of the phagocytosis pathway achieved similar effects as the application of propofol, indicating the involvement of phagocytosis inhibitors in the suppression of phagocytosis by propofol.

## Conclusion

These findings provide preliminary evidence for the potential mechanisms by which propofol regulates microglial phagocytosis and suggests that MFG-E8 is an intermediate target between propofol and phagocytic activity. MFG-E8 may reverse the suppression of phagocytosis by propofol through the regulation of the AMPK and Src signaling pathways.

## Data Availability

The manuscript included all relevant data.

## Abbreviations

CNS: Central nervous system
CRISPR/Cas9: Clustered regularly interspaced short palindromic repeats/CRISPR-associated protein 9
ERK1/2: Extracellular signal regulated kinase-1/2
LPS: Lipopolysaccharide
MFG-E8: Milk fat globule epidermal growth factor 8
OGD: Oxygen-glucose deprivation 9
p38-MAPK: p38-Mitogen-activated protein kinase
p-AMPK: Phosphor-AMP-activated protein kinase
PS: Phosphatidylserine
